# Enhanced Segmentation of Inflamed ROI to Improve the Accuracy of Identifying Benign and Malignant Cases in Breast Thermogram

**DOI:** 10.1155/2021/5566853

**Published:** 2021-04-21

**Authors:** Nirmala Venkatachalam, Leninisha Shanmugam, Genitha C. Heltin, G. Govindarajan, P. Sasipriya

**Affiliations:** ^1^Computer Science and Engineering, St. Joseph's College of Engineering, OMR, Chennai 600 119, India; ^2^School of Computer Science and Engineering, VIT University, Chennai, India; ^3^Surgical Oncology, Harshamitra Super Speciality Cancer Centre and Research Institute, Trichy, India; ^4^Radiation Oncology, Harshamitra Super Speciality Cancer Centre and Research Institute, Trichy, India

## Abstract

Effective analysis of breast thermography needs an accurate segmentation of the inflamed region in Infrared Breast Thermal Images (IBTI) which helps in the diagnosis of breast cancer. However, IBTI suffers from intensity inhomogeneity, overlapping regions of interest, poor contrast, and low signal-to-noise ratio (SNR) due to the imperfect image acquisition process. To mitigate this, this work proposes an enhanced segmentation of the inflamed Region of Interest (ROI) using an active contour method driven by the multiscale local and global fitted image (MLGFI) model. The first phase proposes a bilateral histogram difference-based thresholding (BHDT) method for locating the inflamed ROI. This is then used for automatic initialization of active contours driven by MLGFI to segment the inflamed ROI from IBTI effectively. To prove the effectiveness of this segmentation method, its performance is compared with ground truth image and its accuracy is also evaluated with the state-of-the-art methods (Fuzzy C Means (FCM), Chan-Vese (CV-ACM), and K-means). From the analysis, it is found that the proposed method not only increases the precision and the segmentation accuracy but also reduces the oversegmentation and undersegmentation rate significantly. In the second phase, area-based feature (AF) and average intensity-based feature (AIF) along with the GLCM (gray level cooccurrence matrix) based second-order statistical features are extracted from the inflamed ROI. Based on these features, a system is developed to effectively classify the benign and malignant breast conditions. From the results, it is observed that the proposed model exhibits an improved accuracy of 91.5%, sensitivity of 91%, and specificity of 92% compared to the whole breast thermogram. Hence, it is concluded that the proposed method will improve the efficacy of thermal imaging in the diagnosis of breast cancer.

## 1. Introduction

Breast cancer is still considered as invasive cancer among women worldwide. Almost 2.1 million women are affected by this deadly disease every year which in turn increases the fatality rate of women [1]. According to the survey of 2018, the number of women deceased due to breast cancer is about 627,000 and these rates are increasing globally in all regions [1]. Accurate detection of this disease leads to better treatments and increases the survival rate.

**Figure 1:**
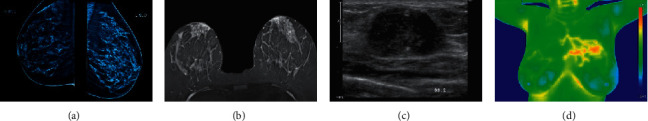
Different imaging modalities: (a) mammography, (b) MRI, (c) ultrasound, and (d) thermography.

Nowadays, many imaging modalities are available for diagnosing these types of diseases. Some of them are mammography, ultrasound, MRI (magnetic resonance imaging), and thermography as shown in Figure 1, different imaging modalities [2–4]. Among them, mammography is still considered as a consistent technique for screening. However, it exhibits some limitations while applying on high-risk women [5–8]. Ultrasound is noninvasive and is suitable for young women, but it purely depends on the expertise of the operator. MRI is expensive and tedious and needs a technical expert for imaging purpose as the false-negative rate is high. All these challenges assert the need for developing new imaging modalities [9].

Infrared thermography has gained more attention over researchers due to its unique characteristics such as noninvasive, nonionizing radiation, painless, and less expensive. It is also highly sensitive and is suitable for all age groups of women/any type of breast condition (dense breast, fibrocystic breast, etc.) [10, 11]. Apart from this, IBTI also measures the temperature variation during the initial stages of the tumour which is caused due to high metabolic activity in the region. It may also be due to angiogenesis which increases the rate of blood flow in the precancerous and cancerous area. The infrared thermal camera captures this temperature distribution by measuring the infrared radiation emitted from the tumour region [5, 6, 10, 11].

Even though imaging of IBTI follows strict protocols and uses advanced thermal cameras, the images exhibit poor contrast, intensity inhomogeneity, overlapping regions of interest, discontinuous boundaries, and low SNR [6, 9] due to imperfection in image acquisition, environmental conditions, lack of skilled technician, and so forth. These limitations have directed the researchers' gaze towards the development of computer-aided analysis tools for reliable and accurate screening.

In medical image analysis, segmentation plays a major role. It assists medical experts to detect the abnormalities of the organs, pathology, visualization in postsurgical planning, diagnosis, treatment for ailments, and so forth [12, 13]. In breast thermography, research mostly focussed on segmentation of the entire breast region and asymmetrical analysis of left and right breast region by differentiating them as normal and abnormal breast [14–18]. However, only a few researchers have analysed the IBTI segmentation based on inflamed ROI. This is because of the challenges over their characteristics like intensity inhomogeneity, overlapping of regions, missing edges, blurriness, noise, poor contrast [6, 19]. However, the inflamed region segmentation identifies the exact location of the malignant region. So, it is evident that the inflamed ROI segmentation will improve the accuracy of identifying benign and malignant cases in IBTI.

Etehadtavakol et al. [19] utilised FCM for segmentation of the hottest region in breast thermal images. Further, bispectral invariant features are extracted from the segmented hot region and classified as malignant, nonmalignant, benign, and normal classes.

Etehadtavakol and Ng [20] compared *k*-means, mean shift, and FCM based clustering algorithms for the segmentation of coloured breast thermogram. FCM performs better than the *k*-means and mean shift.

Golestani et al. [21] implemented ACM based on the CV level set method for segmentation of inflamed ROI in breast thermogram. When compared to the clustering-based methods, it exhibits better results. However, it is not accurate for intensity inhomogeneity images because it considers only the global characteristics of the image, and hence, the initial contour position is not clearly specified.

Milosevic et al. [22] proposed the minimum variance quantization method and morphological operations for inflamed region segmentation in breast thermogram. However, in this method, the parameters were not clearly defined.

In Etehadtavakol et al. [23], the FCM clustering method is used for the segmentation of ROI from breast thermal images. Fractal dimension is calculated for the ROI to differentiate malignant, benign, and normal cases using a limited dataset.

From the comparison of the state-of-the-art methods used in IBTI; region-based ACM performs comparatively better than other methods. Initially, the ACM method utilised gradient information, curvature, image statistics to define the energy function to evolve the curve. Now ACM's are classified into two-category edge-based methods [24–26] and region-based methods [27–37]. Region-based ACMs are further categorized as global [27–29] and local [30, 31] region-based ACM methods. Some of the authors utilised both global and local terms of the image for formulating the energy function which results in an effective segmenting [32–36]. However, many ACM models fail to segment images with inhomogeneous intensity. Consequently, they are highly sensitive to initial contour position. This leads to oversegmentation/undersegmentation. The global region-based methods fail in segmenting the intensity inhomogeneous regions and the local region-based method can segment images only with slight intensity inhomogeneity. Subsequently, the local region-based method examines the local region centred at each pixel point using a fixed scale based on a certain statistical function. At the same time, the fixed scale practise does not segment the image with severe intensity inhomogeneity.

Thus, to effectively segment the severe intensity inhomogeneity images, the scale should be adaptively changed for each local region accordingly. Hence, multiscale local and global region-based analysis idea has to be introduced to effectively segment the inflamed region from IBTI which suffer from severe intensity inhomogeneity. This in turn reduces over- and undersegmentation rate.

Keeping this in view, this work proposed a novel model for the segmentation of the inflamed ROI based on multiscale local and global region-based analysis (MLGFI). In the first phase, the BHDT method is used to locate the inflamed region. The region segmented using BHDT is then used to automatically initialize the contour for the MLGFI model for inflamed ROI segmentation. The result of the proposed inflamed region-based segmentation method is compared with the state-of-the-art methods. In the second phase, based on inflamed ROI segmented, AF and AIF feature along with second-order statistical features are extracted; based on that, a system for identifying benign and malignant breast condition in IBTI is formulated. Finally, the result of the system is evaluated by comparison with the other system that focuses on breast analysis.

The main contribution of this work is as follows:

1. The proposed BHDT method located the inflamed region in IBTI and then used as autoinitialization for the MLGFI, thereby reducing the manual intervention.
2. The proposed MLGFI accurately segments the inflamed ROI in IBTI with severe intensity inhomogeneity.
3. The proposed method reduces both the over- and undersegmentation rate significantly as compared to the state-of-the-art methods.
4. The proposed area and average intensity-based feature along with this GLCM based second-order statistical features are also extracted from segmented inflamed ROI for analysing the IBTI.
5. Developed a system that effectively analyses the IBTI based on the segmented inflamed ROI to identify the benign and malignant condition.

The rest of the paper is organised as follows: Section 2 discusses the proposed dataset for IBTI followed by the proposed method; then, Section 3 discusses the experimental result in comparison with other methods, and finally, Section 4 gives the conclusion.

## 2. Materials and Methods

### 2.1. Data Collection

The IBTI used for this research was collected using noncontact thermography from the “Harshamitra Super Speciality Cancer Centre and Research Institute,” Trichy, Tamil Nadu, India. The infrared camera DITI CX320 with a resolution of 320 × 240 is utilised to capture the image. All standard protocols like imaging in a temperature (20°C–24°C) controlled darkened room with no airflow: the patient was recommended not to have alcohol, smoking, caffeine, and painkillers and to avoid ointments, cream, and so forth, on the area to be tested, before 2 hours of the imaging process, the patient was made to disrobe the top, remove all accessories, and, if required, to bun their hair. The patient was made to sit on the chair with hands placed at the back of the head. The distance between the patient and the thermal camera was kept as one metre. The imaging was taken in 5 different positions like frontal view, left and right lateral view, and left and right oblique view for better analysis purpose. Along with imaging details, the temperature matrix was also provided.

#### 2.1.1. Details of the Proposed Dataset Collected

The IBTI dataset 1 was collected from 50 patients with abnormalities as malignant and benign conditions. The record of each patient like age, history of alcohol and tobacco usage, family history of the disease, and complaints has been collected. Apart from this, clinical finding such as mammogram or ultrasound or both, thermogram and fine-needle aspiration (FNAC) if needed, has been collected. If the patient is positive for carcinoma, then the patient was subjected to a biopsy test as well.  Table 1 displays the patient details. The proposed work was also implemented on a public dataset available at the visual lab, Fluminense Federal University, Brazil (DMR-IR) [38] (i.e., Dataset 2). 50 images with malignant and benign cases were considered for this work from this dataset. These images were captured by following the standard protocols, using FLIR infrared camera with a pixel resolution of 640 × 480 pixels and temperature sensitivity of 0.04°C. The details of patients including images at 5 different positions, temperature matrix, clinical findings, mammogram, ultrasound, and biopsy details have been collected from the patients.

### 2.2. Segmentation of Inflamed ROI

#### 2.2.1. Preprocessing

**Figure 2:**
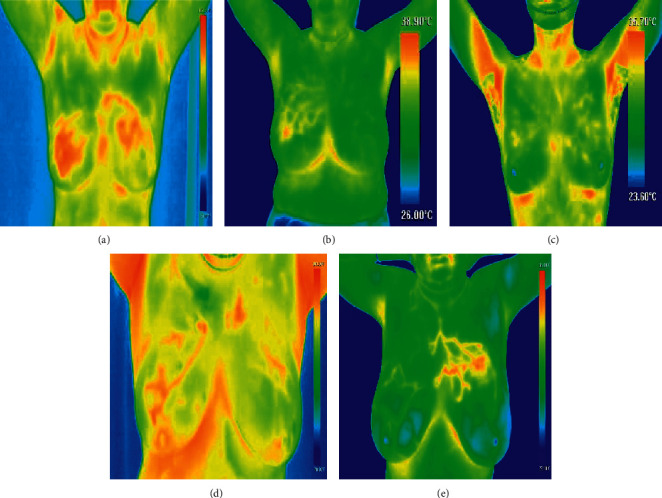
Images of different focal lengths: (a, b, c) patients with too far focal length, (d) patient with too close focal length, and (e) patient with a correct focal length.

The dataset 1 collected contains diverse images, taken in different focus and imaging properties, as seen in Figure 2, each image has a different focus and zoom. So, preprocessing is done for eliminating the nonbreast region which makes all the images look evenly. Also, the removal of the nonbreast region will reduce the computational complexity. It also limits the next progressing step to the particular region [39]. Two sets of ground truth images have been created with help of experts for the datasets. First set contains the boundary area of the breast created for all cases of breast thermogram and the second set of the ground truth contains only the inflamed region segmented in the IBTI.

**Figure 3:**
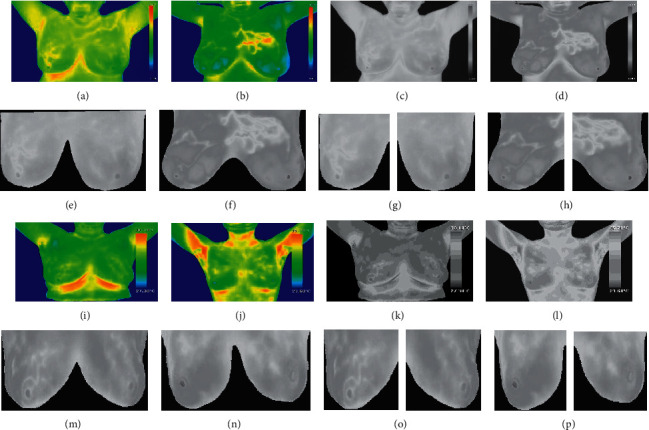
Preprocessing steps of the malignant cases its corresponding (a, b) pseudocoloured image, (c, d) grayscale image, (e, f) nonbreast area removed image, (g, h) left and right breast separated image and benign cases its corresponding (i, j) pseudocoloured image, (k, l) grayscale image, (m, n) nonbreast area removed image, and (o, p) left and right breast separated image.

Initially, the pseudocoloured images are converted to a grayscale image; then, they are multiplied with the first ground truth mask to eliminate the nonbreast region. Since ground truth was segmented with the help of experts, the eliminated nonbreast region with ground truth will be more accurate than manual cropping. Then, the left and right breast is segmented by dividing the image exactly in the centre.  Figure 3 shows the steps involved in preprocessing.  Figure 3 shows the malignant cases, 3(a) and 3(b) are the pseudocoloured images, 3(c) and 3(d) show its grayscale image, 3(e) and 3(f) show the nonbreast area removed image, and last 3(g) and 3(h) show the left and right breast separated image. Similarly, the benign cases 3(i) and 3(j) show the pseudocoloured image, 3(k) and 3(l) show the grayscale image, 3(m) and 3(n) show the nonbreast area removed image, and 3(o) and 3(p) show the left and right breast separated image.

#### 2.2.2. Locating the Inflamed Region Using the BHDT Method

IBTI suffers from severe intensity inhomogeneity; that is, the intensity distribution between the affected and nonaffected breast varies significantly. This can be easily differentiated using a histogram. Hence, global thresholding based on the bilateral histogram is proposed for locating the inflamed region in IBTI. Thus, in case of abnormal IBTI, the bilateral intensity between affected and nonaffected breast (left and right) varies largely. While implementing the general global thresholding, the entire image to identify the threshold value, the histogram taken for the entire image fails in identifying this bilateral intensity difference. Hence, to overcome this, the proposed work utilises a technique called BHDT for locating the inflamed regions in IBTI.

**Figure 4:**
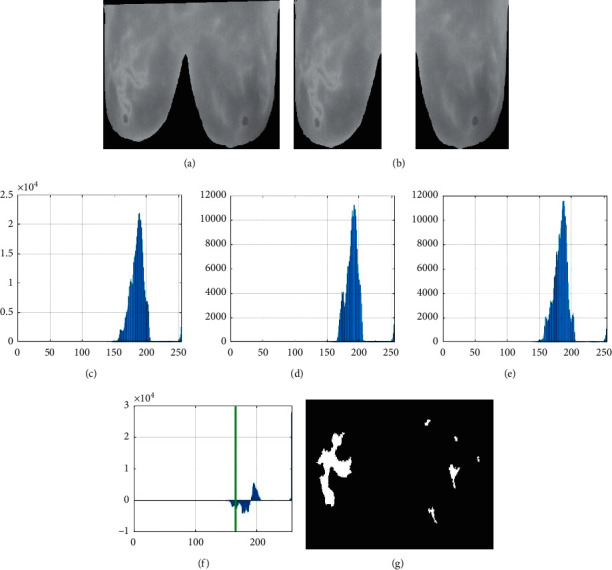
(a) Breast area cropped, (b) left and right breast separated, (c) original histogram, (d) histogram of right breast, (e) histogram of left breast, (f) bilateral histogram difference of left and right breast, and (g) after BHDT.

In BHDT, the histogram is plotted separately for the left breast and right breast, their bilateral histogram difference calculated is shown in Figure 4(f), and the final threshold value is selected. Using this threshold, global thresholding is done on the IBTI and the inflamed region is located.

Here, the inflamed region got from BHDT is not accurately segmented especially in the boundary of inflamed ROI. BHDT fails in segmenting the boundary where the intensity inhomogeneity is severe.

So, MLGFI is proposed to segment the severe intensity inhomogeneous region and to segment the inflamed ROI accurately. This located inflamed region from BHDT is automatically made as an initial contour for the MLGFI model. This avoids the need for a manual interruption in the initialization of contour and thus reduces the computational time.

#### 2.2.3. Multiscale Local and Global Image Fitted Model

IBTI suffers from severe intensity inhomogeneity which may be due to imperfect image acquisition process and environmental conditions. The proposed method uses MLGFI for accurate segmentation of inflamed ROI. This results in a decreased rate of over and undersegmentation significantly. Intensity inhomogeneity is a smooth spatially varying function that changes the intensity value of the images, which would be constant for the particular region. It tends to have overlap between regions and thus increases the complexity of differentiation between inflamed ROI and other hot regions. Thus, the presence of inhomogeneity will reduce the accuracy of inflamed ROI segmentation in IBTI and causes oversegmentation. The most common model to describe the intensity inhomogeneity affected image is given by [32, 37]

(1) $$\begin{array}{c} I\left(x\right)=b\left(x\right)J\left(x\right)+n\left(x\right). \end{array}$$

In equation (1), let *I*(*x*) : Ω⟶*R*^2^ be an image with intensity inhomogeneity, *J*(*x*) be the restored image without intensity inhomogeneity, *b*(*x*) denote the intensity inhomogeneity, and *n*(*x*) be the additive noise [32, 39]. Generally, the spectrum of intensity inhomogeneity is concentrated in the lower frequency band. The local region-based method examines the local region centred at each pixel point using a fixed scale based on a statistical function [40, 41]. The fixed scale practise for all local regions segments the image with only moderate-intensity inhomogeneity. To effectively segment the images with severe intensity inhomogeneity for each local region, the scale should adaptively change. Since the intensity inhomogeneity spectrum is concentrated on the lower frequency band, multiscale low pass filter is used to select the lower frequency artefact effectively over the high frequency. Hence, a multiscale mean filter is tailored to determine the local circular region centred for each pixel *x* in a given image *I* [41]:

(2) $$\begin{array}{c} {\text{MSMF}}_{i}\left(x\right)=\frac{1}{n}\sum_{y\in R_{x,i}}I\left(y\right), \end{array}$$

where MSMF_*i*_(*x*) is the multiscale mean filter with subscript *i* the radius of the circular region (scale) and *n* is the number of pixels and local regions *R*_*x*,*i*_. The multiscale local intensity information equation (3) is calculated by taking the mean of the previous equation [41]:

(3) $$\begin{array}{c} M_{k}\left(x\right)=\frac{1}{k}\sum_{i=1}^{k}{\text{MSMF}}_{i}\left(x\right), \end{array}$$

where *k* is the number of scales. The value *k* should be neither too low nor too high. A low value will reduce the number of the local circular region to be analysed for each pixel. Similarly, the high value will increase the number of the local circular region to be analysed for each pixel, which in turn will increase the computational cost. Thus, careful considerations have to be taken while choosing the *k* value. Based on the chosen value, the computational cost will vary.

The logarithmic transform of equation (1) is given as

(4) $$\begin{array}{c} \log\;I\left(x\right)=\log\;b\left(x\right)+\log\;J\left(x\right). \end{array}$$

To simplify the computation, the noise term is eliminated. Since the intensity inhomogeneity is considered as a low-frequency artefact, *b*(*x*) is replaced with multiscale local information term *M*_*k*_(*x*). The approximation of intensity inhomogeneity free image *J* is given as

(5) $$\begin{array}{c} \log\;\hat{j}\left(x\right)=\log\;I\left(x\right)-\log\;M_{k}\left(x\right)+\log\;C_{N}, \end{array}$$

where *C*_*N*_ is a normalized constant to preserve the mean intensity of *J*. For simplifying the computation equation (5), it can be rewritten as [41]

(6) $$\begin{array}{c} \hat{j}\left(x\right)=\frac{I\left(x\right)C_{N}}{M_{k}\left(x\right)}. \end{array}$$

Thus, the approximation of intensity inhomogeneity image $\hat{j}$ (*x*) can be achieved from the ratio of the normalized weighted component of image *I*(*x*)*C*_*N*_ with the multiscale local intensity information *M*_*k*_(*x*). Hence, the images with severe intensity inhomogeneity can be segmented.

To segment the inflamed ROI accurately in IBTI with severe intensity inhomogeneity, overlapping regions, weak boundaries, the level set based on the local and global fitted image is approximated with the $\hat{j}$ (*x*). The energy function of the multiscale local and global fitted image model is given as

(7) $$\begin{array}{c} E_{\text{MLGFI}}=E_{D}+E_{R}. \end{array}$$

The regularization term includes both arc length penalty term and reinitialization penalty term [42]:

(8) $$\begin{array}{c} E_{R}=\mu \int_{\Omega }{\left|\nabla H_{\varepsilon }\left(\phi \right)\right|}^{2}\text{d}x+v\int_{\Omega }{\left(\nabla \left(\phi \left(x\right)-1\right)\right)}^{2}\text{d}x. \end{array}$$

The data term of the multiscale local and global fitted image is given as

(9) $$\begin{array}{c} E_{D}=\int_{\Omega }\left(\frac{I\left(x\right)C_{N}}{M_{k}\left(x\right)}-I_{\text{LFI}}\left(x\right)\right)\left(I\left(x\right)-I_{\text{GFI}}\left(x\right)\right)\text{d}x, \end{array}$$

(10) $$\begin{array}{c} E_{D}=\;\int_{\Omega }\left(\frac{I\left(x\right)C_{N}}{M_{k}\left(x\right)}\right)-\left(f_{1}\left(x\right)M_{1}+f_{2}\left(x\right)M_{2}\right)\left(I\left(x\right)-\left(c_{1}M_{1}+\;c_{2}M_{2}\right)\right)\text{d}x, \end{array}$$

where *f*_1_(*x*) and *f*_2_(*x*) are the local intensity means and *c*_1_ and *c*_2_ are the global intensity means. *M*_1_= *H*_*ε*_(*ϕ*) and *M*_2_=(1 − *H*_*ε*_(*ϕ*)), and the Heaviside function *H*_*ε*_(*ϕ*)=(1/2)+(1/*π*)arctan(*ϕ*/*ε*):

(11) $$\begin{array}{c} c_{1}=\;\frac{\int_{\Omega }I\left(x\right)H_{\varepsilon }\left(\phi \left(x\right)\right)\text{d}x}{\int_{\Omega }H_{\varepsilon }\left(\phi \left(x\right)\right)\text{d}x},\\ c_{2}=\;\frac{\int_{\Omega }I\left(x\right)\left(1-H_{\varepsilon }\left(\phi \left(x\right)\right)\text{d}x\right)}{\int_{\Omega }\left(1-H_{\varepsilon }\left(\phi \left(x\right)\right)\text{d}x\right)},\\ f_{1}\left(x\right)=\;\frac{\int_{\Omega }K_{\sigma }\left(x\right)*\left[I\left(x\right)C_{N}/M_{k}\left(x\right)M_{1}\left(\phi \left(x\right)\right)\right]}{\int_{\Omega }K_{\sigma }\left(x\right)*M_{1}\left(\phi \left(x\right)\right)},\\ f_{2}\left(x\right)=\;\frac{\int_{\Omega }K_{\sigma }\left(x\right)*\left[\;I\left(x\right)C_{N}/M_{k}\left(x\right)M_{2}\left(\phi \left(x\right)\right)\right]}{\int_{\Omega }K_{\sigma }\left(x\right)*M_{2}\left(\phi \left(x\right)\right)}. \end{array}$$

Finally, the energy function in equation (10) is minimized using the steepest gradient descent method. Keeping the values of *c*_1_, *c*_2_, *f*_1_, and *f*_2_ fixed, the minimization of energy function with respect to *ϕ* the Euler–Lagrange equation for *ϕ* can be deduced as

(12) $$\begin{array}{c} \frac{\partial \phi }{\partial t}=\delta_{\varepsilon }\left(\phi \right)\left(\left(\frac{I\left(x\right)C_{N}}{M_{k}\left(x\right)}-I_{\text{LFI}}\left(x\right)\right)\left(f_{1}-f_{2}\right)+\left(I\left(x\right)-I_{\text{GFI}}\left(x\right)\right)\left(c_{1}-c_{2}\right)\right)+\mu \delta_{\varepsilon }\left(\phi \right)\text{div}\left(\frac{\nabla \phi }{\left|\nabla \phi \right|}\right)+v\left(\nabla^{2}\left(\phi \right)-\text{div}\left(\frac{\nabla \phi }{\left|\nabla \phi \right|}\right)\right). \end{array}$$

Finally, an automatic stopping condition is executed. It can be also carried out manually, but it requires manual interruption every time to set the number of iterations “*N*.” This will cause oversegmentation and also increases the computational costs. To avoid these setbacks, an automatic stopping condition is introduced. From the results, it is found that the level set function *ϕ* becomes stationary when the curve reaches its desired boundary. Thus, based on these observations, the stopping condition is evaluated. For every iteration, the output image is converted into binary image *I*_*b*_. Once the *ϕ* reached its desired boundary, the evolution of contour stops. *I*_*b*_ value will be the same for the current and the next iteration. During execution, the iteration stops when the difference between the current *I*_*b*(*k*)_ and the previous *I*_*b*(*k*−1)_ reaches zero By choosing the ideal stopping criterion, oversegmentation and computational costs can be reduced.

The algorithm for segmentation of inflamed ROI using MLGFI is discussed below (Algorithm 1).

**Figure 5:**
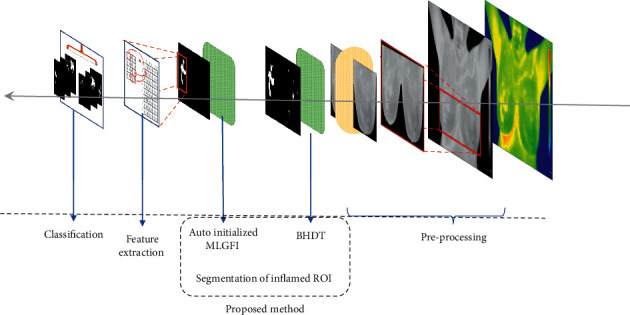
An analysis system of IBTI.

**Figure 6:**
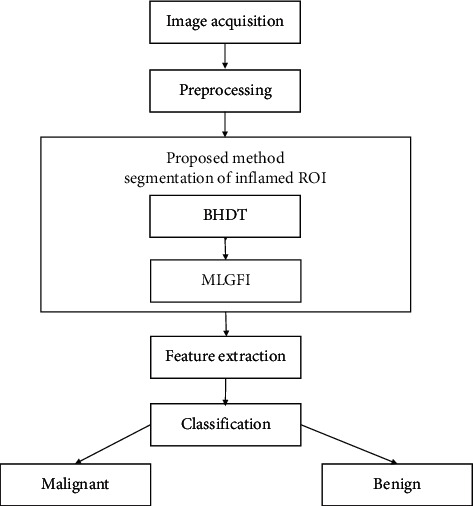
Block diagram of system to identify malignant and benign cases in IBTI.

To classify the malignant and benign breast condition accurately, an analysis system based on inflamed ROI segmented from IBTI is implemented and the same is depicted in Figures 5 and 6.

### 2.3. Feature Extraction and Classification

Feature extraction plays a significant role in image processing. An effective feature extracted will lead to an accurate analysis of the image. From the clear study of IBTI, it shows the area of cancer region of malignant IBTI is wider and its average intensity is also high. Based on this study, two features of AF and AIF are formulated using area and average intensity values. In this process, the segmented image is converted to a binary image, where white pixel corresponds to the inflamed ROI with value “1” and black pixels correspond to the residual region with value “0.” The white pixel count corresponds to the Area of Inflamed ROI (AIR) and the black pixel count corresponds to the Area of Residual Region (ARR). The absolute difference between AIR and ARR formulates AF:

(13) $$\begin{array}{c} \text{AF}=\left|\text{AIR}-\text{ARR}\right|. \end{array}$$

Similarly, the white pixel average intensity value corresponds to the Average Intensity of Inflamed ROI (AIIR) and the black pixel average intensity value corresponds to the Average Intensity of Residual Region (AIRR). From this, AIF can be calculated as

(14) $$\begin{array}{c} \text{AIF}=\left|\text{AIIR}-\text{AIRR}\right|. \end{array}$$

This absolute value of the area and average intensity features shows a significant difference between the malignant and benign cases.

Along with this, the second-order statistical features based on the cooccurrence matrix, GLCM is used in this work [42]. GLCM is considered one of the best feature extraction techniques, which helps in calculating some best textural properties to analyse an image. A total of twenty-one second-order statistical features [42–44] are extracted using a cooccurrence matrix from the inflamed region and feature vectors are calculated for all 23 features.

Support Vector Machine (SVM) is employed for classification purpose [45, 46]. SVM is the most familiar method which is used in many pattern recognition problems especially widely used in binary classification problems. The twenty-three feature vectors extracted are used to train the SVM model. SVM using Radial Basis Function (RBF) kernel was used in this work. Among 50 images from dataset 1 and 50 images from dataset 2, 60% of these images from both datasets were used to train the model and the remaining 40% for testing.

## 3. Experiment Results and Discussion

In this section, the evaluation metrics of the proposed method are discussed for both datasets. First, the segmentation of the inflamed ROI (proposed work) is evaluated and compared with the state-of-the-art methods, and then the identification of benign and malignant cases from IBTI is carried out.

### 3.1. Evaluation of Inflamed ROI Segmentation Based on Qualitative and Quantitative Analysis

The proposed segmentation of the inflamed ROI is compared with the ground truth image. Here, the second set ground truth mask is considered, which contains the segmented inflamed ROI. The ground truth generated should be very accurate. In this work, manual ground truth is generated with the help of experts (more than one) in the same domain. Thus, two sets of ground truth are generated for both datasets 1 and 2, respectively.

**Figure 7:**
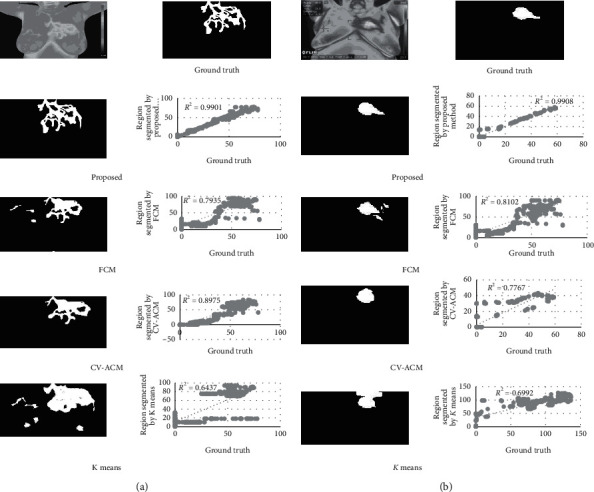
(a) Image 1 (dataset 1): corresponding greyscale image, ground truth, segmentation result of the proposed method with linear regression plot iteration: 48, CPU time: 5.468s; FCM method with linear regression plot iteration: 78, CPU time: 15.703s; CV-ACM method with linear regression plot iteration: 290, CPU time: 11.76s; K-means method with linear regression plot iteration: 103, CPU time: 17.73s. (b) Image 2 (dataset 2): corresponding greyscale image, ground truth, segmentation result of the proposed method with linear regression plot iteration: 40, CPU time: 5.093s; FCM method with linear regression plot iteration: 71, CPU time: 12.093s; CV-ACM method with linear regression plot iteration: 260, CPU time: 10.64s; and K-means method with linear regression plot iteration: 100, CPU time: 15.34s.

Performance evaluation of the proposed method of segmenting the inflamed ROI is carried out by comparing the results of the proposed method with the ground truth and the linear regression model is depicted in Figure 7. The proposed method is also compared with the state-of-the-art methods like FCM [20], CV-ACM [21], and K-means [47]. From Figure 7, it is observed that the linear regression plot analysis has been carried out for the proposed method. *R*^2^ is the correlation of determination, which indicates exactly how many points fall on the regression line (compared with ground truth). If the *R*^2^ value is above 0.95 (i.e., 95%), then it is considered as a good fit. Hence, from Figure 7, it is noticed that that the *R*^2^ value of the proposed method is 0.9701 and 0.9808. Hence, it is concluded that the proposed topology exhibits good fit compared other methods. Similarly, from Figure 7, it is also observed that the number of iterations and CPU time of the proposed method is significantly lower than other methods.

Further, segmentation accuracy (SA), similarity measures like Jaccard Index (JI), and dice similarity (DS) [48] are evaluated to measure the efficiency of the proposed topology. Thus, for better segmentation, the value of SA, JI, and DS should be maximum. Apart from this, Undersegmentation Rate (USR) and Oversegmentation Rate (OSR) are also examined. USR is the proportion of unsegmented inflamed ROI and OSR is the proportion of segmented noninflamed area [49]. Both USR and OSR values should be low for perfect segmentation.

**Figure 8:**
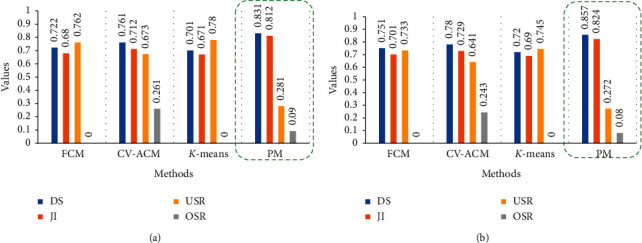
Performance of the metrics of the proposed method with state-of-the-art methods: (a) Dataset 1. (b) Dataset 2.

Table 2 depicts the metrics used for performance evaluation of the proposed method. Here, *X* is the segmented inflamed ROI and *Y* is the ground truth of the inflamed ROI.  Table 3 and Figure 8 show the comparison of the performance of the proposed inflamed ROI segmentation with the state-of-the-art methods using SA, DS, JI, OSR, and USR.

From the comparative study, it is clear that the results of the proposed method are better than the state-of-the-art methods in case of both datasets. The SA of the proposed method for both datasets is 91.2 and 92.4, respectively, which is significantly better than other methods. The DS and JI values of the proposed method for both the datasets are 0.831, 0.812 and 0.857, 0.824, respectively, which is encouragingly higher than the other methods. Also, the OSR and USR values of the proposed method for both datasets (0.09, 0.281, and 0.08, 0.272) are low when compared to other methods. High OSR values will decrease the size of inflamed ROI segmented compared to the ground truth and similarly, high USR values will increase the size of the inflamed ROI segmented when compared with the ground truth. FCM and *K*-means methods have better OSR values, but they perform significantly lower in all other metrics in comparison with the proposed method. Hence, it is concluded that the proposed model exhibits more accurate and reliable segmentation of inflamed ROI than all the other models depicted in Table 3.

However, in few cases, it is observed that the proposed method fails in segmenting the inflamed ROI and thereby increasing the undersegmentation rate. This is because of the unclear boundary or edges of the ROI. In the future, this limitation can be mitigated by incorporating the edge-based term along with the region-based ACM to avoid the boundary leakage.

### 3.2. Evaluation of the Entire IBTI Analysis System

A total of 100 images with both malignant and benign cases were considered as a test system. Out of this, 60% of images were considered as training dataset and the remaining 40% were considered as test dataset. 21 second-order statistical feature vector and additional AF and AIF features are extracted from segmented inflamed ROI and are fed to the SVM-RBF classifier. The optimal C and gamma were chosen using grid search, with C ranging from 10^−1^ to 10^3^ and gamma ranging from 10^0^ to 10^−4^. Finally, an accuracy of 91.5% is achieved with gamma = 0.01 and *C* = 1. To validate the classifier performance, the standard measures like accuracy, sensitivity, and specificity along with positive predictive value (PPV) and negative predictive value (NPV) [6] are considered. The four metrics used for the above measures calculation are True Positive (TP), True Negative (TN), False Positive (FP), and False Negative (FN) which are got from the confusion matrix.

The expressions for the measures are given as follows:

(15) $$\begin{array}{c} \text{Accuracy}=\frac{\text{TP}+\text{TN}}{\text{TP}+\text{TN}+\text{FP}+\text{FN}},\\ \text{Sensitivity}=\frac{\text{TP}}{\text{TP}+\text{FN}},\;\\ \text{Specificity}=\frac{\text{TN}}{\text{TN}+\text{FP}},\\ \text{PPV}=\frac{\text{TP}}{\text{TP}+\text{FP}},\\ \text{NPV}=\frac{\text{TN}}{\text{TN}+\text{FN}}. \end{array}$$

**Figure 9:**
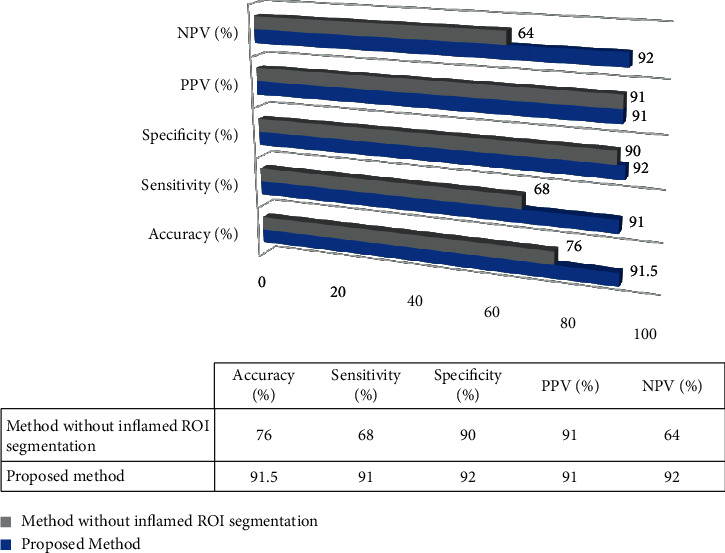
IBTI analysis system metrics.

The IBTI analysis system based on the proposed method for segmentation of inflamed ROI has resulted in better accuracy 91.5%, sensitivity 91%, specificity 92%, PPV 91%, and NPV 92%. To evaluate the efficacy of the proposed method, the comparison is made by extracting the same set of features from each IBTI without inflamed ROI segmentation as shown in Figure 9. From Figure 9, it is found that IBTI with the proposed method outperforms the other methods without segmentation of inflamed ROI.

## 4. Conclusion

Inflammatory regions are the first indication of abnormalities in breast thermograms. Precise segmentation of this ROI will aid in the proper diagnosis of breast cancer. Hence, this work focuses on the segmentation of the inflamed ROI using MLGFI. In the proposed method, the inflamed region is located first using BHDT which is then used to automatically initialize the MLGFI. In BHDT, the histogram is plotted separately for the left and right breast and their bilateral histogram difference is calculated; on basis of this, the global thresholding has arrived. This avoids the need for a manual interruption in ACM to initialize the contour and also reduces the computational time. The output from BHDT is then used to initialize the MLGFI. This method was found to handle the severe intensity inhomogeneity of IBTI and was able to precisely segment the inflamed ROI by decreasing the oversegmentation and undersegmentation rate significantly. Secondly, AF and AIF along with GLCM based second-order statistical features are extracted from the segmented inflamed ROI. From these extracted features, the malignant or benign breast condition from IBTI is identified. Thus, the accuracy of the proposed inflamed ROI segmentation is evaluated using two datasets and is also compared with the other state-of-the-art methods to prove its significance. From the analysis, it is found that the SA of the proposed method for both datasets is about 91.2% and 92.4% and is significantly better than the state-of-the-art methods.

Finally, the accuracy, sensitivity, and specificity of the proposed classifier are also analysed. From the obtained results, it is evident that the identification of abnormalities using the proposed segmentation method of the inflamed ROI is more reliable than the whole breast analysis. Thus, the measured metrics, such as accuracy (91.5%), sensitivity (91%), and specificity (92%), demonstrate the effectiveness, reliability, and accuracy of the proposed system in the diagnosis of breast cancer using thermography. Future developments could be exploring the use of deep learning in classification.

## Figures and Tables

**Algorithm 1:**
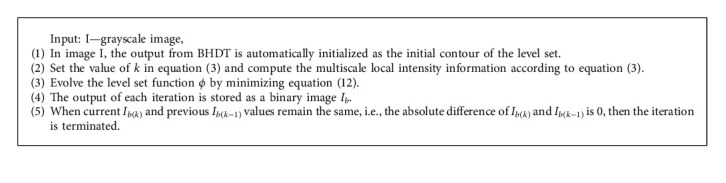
Algorithm for proposed MLGFI-based inflamed ROI segmentation.

**Table 1:** Details of the patient.

S. number	Age	Family history	Tobacco	Alcohol	Complaint	Clinical findings	Mammo	Mammo LOC	ULtra	Ultra LOC	Thermo	FNAC	Biopsy	Biopsy LOC	Treatment
*Malignant breast condition*															
1	45	No	No	No	Rt-BL	Rt-BL	MS	Rt-OUQ	MS	Rt-OUQ	Rt-IRH, SBL	Rt-MS	IDC- Grade II	Rt-OUQ	MRM
2	55	No	No	No	Lt- BL	Lt- BL	MS	Lt-OUQ	MS	Lt-OUQ	Lt-IRH, SBL	Lt-MS	IDC- Grade II	Lt-OUQ	MRM
3	53	No	No	No	Lt- BL	Lt- BL	MS	Lt-OLQ	MS	Lt-OLQ	Lt-IRH, SBL	Lt-MS	IDC- Grade II	Lt-OLQ	MRM
4	35	No	No	No	Rt-BL	Rt-BL	MS	Rt-OLQ	MS	Rt-OLQ	Rt-IRH, SBL	Rt-MS	INDC- Grade III	Rt-OLQ	MRM
5	49	No	No	No	Lt-BL	Lt-BL	MS	Lt- CP	MS	Lt- CP	Lt-IRH, SBL	Lt-MS	IDC- Grade II	Lt- CP	MRM
6	35	No	No	No	Rt-BL	Rt-BL	MS	Rt-OUQ	MS	Rt-OUQ	Rt-IRH, SBL	Rt-MS	INDC- Grade II	Rt-OUQ	MRM
7	44	No	No	No	Rt-BL	Rt-BL	MS	Rt-IUQ	MS	Rt-IUQ	Rt-IRH, SBL	Rt-MS	IDC- Grade II	Rt-IUQ	MRM
8	41	No	No	No	Rt-BL	Rt- BL	MS	Rt-OUQ	MS	Rt-OUQ	Rt-IRH, SBL	Rt-MS	INDC- Grade II	Rt-OUQ	MRM
9	42	No	No	No	Rt-BL	Rt-BL	MS	Rt-OLQ	MS	Rt-OLQ	Rt-IRH, SBL	Rt-MS	IDC- Grade II	Rt-OLQ	MRM
10	45	Yes	No	No	Lt-BL	Lt-BL	MS	Lt-OUQ	MS	Lt-OUQ	Lt-IRH, SBL	Lt-MS	IDC- Grade II	Lt-OUQ	MRM
11	37	No	No	No	Rt-BL	Rt-BL	MS	Rt-OUQ	MS	Rt-OUQ	Rt-IRH, SBL	Rt-MS	IDC- Grade II	Rt-OUQ	MRM
12	51	No	No	No	Lt-BL	Lt-BL	MS	Lt-OUQ	MS	Lt-OUQ	Lt-IRH, SBL	Lt-MS	IDC- Grade II	Lt-OUQ	MRM
13	55	No	No	No	Rt-BL	Rt-BL	MS	Rt-OLQ	MS	Rt-OLQ	Rt-IRH, SBL	Rt-MS	IDC- Grade II	Rt-OLQ	MRM
14	53	No	Yes	No	Rt-BL	Rt-BL	MS	Rt-OLQ	MS	Rt-OLQ	Rt-IRH, SBL	Rt-MS	IDC- Grade II	Rt-OLQ	MRM
15	56	No	No	No	Lt-BL	Lt-BL	MS	Lt-OLQ	MS	Lt-OLQ	Lt-IRH, SBL	Lt-MS	IDC- Grade II	Lt-OLQ	MRM
16	51	No	No	No	Rt-BL	Rt-BL	MS	Rt-OUQ	MS	Rt-OUQ	Rt-IRH, SBL	Rt-MS	IDC- Grade II	Rt-OUQ	MRM
17	60	No	Yes	No	Rt-BL	Rt-BL	MS	Rt-IUQ	MS	Rt-IUQ	Rt-IRH, SBL	Rt-MS	IDC- Grade II	Rt-IUQ	MRM
18	42	No	No	No	Rt-BL	Rt-BL	MS	Rt-OLQ	MS	Rt-OLQ	Rt-IRH, SBL	Rt-MS	IDC- Grade II	Rt-OLQ	MRM
19	39	No	No	No	Rt-BL	Rt-BL	MS	Rt-OUQ	MS	Rt-OUQ	Rt-IRH, SBL	Rt-MS	IDC- Grade II	Rt-OUQ	MRM
20	70	Yes	No	No	Lt-BL	Lt-BL	MS	Lt- IUQ	MS	Lt- IUQ	Lt-IRH, SBL	Lt-MS	IDC- Grade II	Lt- IUQ	MRM
21	58	No	No	No	Lt-BL	Lt-BL	MS	Lt-OUQ	MS	Lt-OUQ	Lt-IRH, SBL	Lt-MS	IDC- Grade II	Lt-OUQ	MRM
22	48	No	No	No	Rt-BL	Rt-BL	MS	Rt-OLQ	MS	Rt-OLQ	Rt-IRH, SBL	Rt-MS	IDC- Grade II	Rt-OLQ	MRM
23	49	No	No	No	Both-BP	Lt-BL	MS	Lt-IUQ	MS	Lt-IUQ	Lt-IRH, SBL	Lt-MS	IDC- Grade II	Lt-IUQ	MRM
24	48	No	No	No	Rt-BL	Rt-BL	MS	Rt-IUQ	MS	Rt-IUQ	Rt-IRH, SBL	Rt-MS	IDC- Grade II	Rt-IUQ	MRM
25	42	Yes	No	No	Lt-BL	Lt-BL	MS	Lt-OUQ	MS	Lt-OUQ	Lt-IRH, SBL	Lt-MS	IDC- Grade II	Lt-OUQ	MRM
26	51	No	No	No	Rt-BL	Rt-BL	MS	Rt-OLQ	MS	Rt-OLQ	Rt-IRH, SBL	Rt-MS	IDC- Grade II	Rt-OLQ	MRM
27	65	No	No	No	Lt-BL	Lt-BL	MS	Lt-IUQ	MS	Lt-IUQ	Lt-IRH, SBL	Lt-MS	IDC- Grade II	Lt-IUQ	MRM
28	86	No	No	No	Rt-BL	Rt-BL	MS	Rt-OUQ	MS	Rt-OUQ	Rt-IRH, SBL	Rt-MS	IDC- Grade II	Rt-OUQ	MRM
29	62	No	No	No	Lt-BL	Lt-BL	MS	Lt-OLQ	MS	Lt-OLQ	Lt-IRH, SBL	Lt-MS	IDC- Grade II	Lt-OLQ	MRM
30	62	No	No	No	Lt-BL	Lt-BL	MS	Lt- CP	MS	Lt- CP	Lt-IRH, SBL	Lt-MS	IDC- Grade II	Lt- CP	MRM
31	40	Yes	No	No	Lt-BL	Lt-BL	MS	Lt-IUQ	MS	Lt-IUQ	Lt-IRH, SBL	Lt-MS	IDC- Grade II	Lt-IUQ	MRM
32	30	No	No	No	Lt-BL	Lt-BL	MS	Lt-IUQ	MS	Lt-IUQ	Lt-IRH, SBL	Lt-MS	IDC- Grade II	Lt-IUQ	MRM
33	35	No	No	No	Lt-BL	Lt-BL	MS	Lt-IUQ	MS	Lt-IUQ	Lt-IRH, SBL	Lt-MS	IDC- Grade II	Lt-IUQ	MRM
34	37	No	No	No	Rt-BL	Rt-BL	MS	Rt- IUQ	MS	Rt- IUQ	Rt-IRH, SBL	Rt-MS	IDC- Grade II	Rt- IUQ	MRM

*Benign breast condition*															
35	19	No	No	No	Both-BL	Both-FCC	Both-BS	__	Both-BS	__	Both-H	FD	__	__	RL
36	33	No	No	No	Rt-BL	Rt-FCC	Rt-BS	__	Rt-BS	__	Rt-H	FD	__	__	RL
37	28	No	No	No	Both-BL	Both-FCC	Both-BS	__	Both-BS	__	Both-H	FD	__	__	RL
38	39	No	No	No	Both-BL	Both-FCC	Both-BS	__	Both- BS	__	Both-H	FD	__	__	RL
39	56	No	No	No	Lt-BP, BL	Lt-FCC	Lt-BS	__	Lt-BS	__	Lt-H	FD	FD	__	RL
40	38	No	No	No	Both-BL	Both-FCC	Both- BS	__	Both- BS	__	Both-H	FD	FD	__	RL
41	32	No	No	No	Rt-BL	Rt-BL	Rt-BS	__	Rt- BS, BL	__	Rt-H	FD	__	__	RL
42	41	No	No	No	Rt-BL	Rt- BL	Rt-BS	__	Rt- BS, BL	__	Rt-H	FD	FD	__	RL
43	38	No	No	No	Lt-BP, BL	Lt-FCC	Lt-BS	__	Lt-BS	__	Lt-H	FD	FD	__	RL
44	16	No	No	No	Rt-BL	Rt-BL	Rt-BS	__	Rt-BS	__	Rt-H	FD	__	__	RL
45	49	No	No	No	Lt-BL	Lt-FCC	Lt-BS	__	Lt-BS	_	Lt-H	FD	__	__	RL
46	50	No	No	No	Lt-BL	Lt-BL	Lt-BS	__	Lt-BS	__	Lt-H	FD	__	__	RL
47	52	No	No	No	Lt-BL	Lt-BL	Lt-BS	__	Lt-BS	__	Lt-H	FD	FD	__	RL
48	64	No	No	No	Lt-BL	Lt-BL	Lt-BS	__	Lt-BS	__	Lt-H	FD	FD	__	RL
49	37	No	No	No	Rt-BL	Rt-BL	Rt-BS	__	Rt-BS	__	Rt-H	FD	FD	__	RL
50	48	No	No	No	Rt-BL	Rt-BL	Rt-BS	__	Rt-BS	__	Rt-H	FD	FD	__	RL

FH: family history, CF: clinical findings, Mammo: mammogram, Ultra: ultrasound, FNAC: fine-needle aspiration cytology, BL: breast lesion, BP: breast pain, Rt: right breast, Lt: left breast, FCC: fibrocystic change, FD: fibrocystic disease, BS: benign suggested, MS: malignancy suggested, OUQ: outer upper quadrant, OLQ: outer lower quadrant, IUQ: inner upper quadrant, ILQ: inner lower quadrant, CP: central position, MP: mensural problem, IRH: irregular hyperthermic, H: hyperthermic, SBL: suspicious breast lesion, IDC: invasive ductal carcinoma, INDC: infiltrating duct carcinoma, and LOC: location.

**Table 2:** Metrics used for performance evaluation of inflamed ROI segmentation.

Metrics	Formulas with range	Value of accurate segmentation
SA [6]	*X*∩*Y*/*X* ∪ *Y*, (0–100)	100
DS [48]	2| *X*∩*Y*|/| *X* |+| *Y* |, (0-1)	1
JI [48]	| *X* ∩*Y*|/| *X* ∪ *Y*|, (0-1)	1
USR [49]	|*Y* − (*X*∩*Y*)|/|*Y*|, (0-1)	0
OSR [49]	|*X* − (*X*∩*Y*)|/|*X*|, (0-1)	0

**Table 3:** Comparison of the metrics of the proposed method with state-of-the-art methods.

Methods	Dataset 1					Dataset 2				
	SA (%)	DS	JI	OSR	USR	SA (%)	DS	JI	OSR	USR
PM	91.2	0.831	0.812	0.09	0.281	92.4	0.857	0.824	0.08	0.272
FCM [20] 2017	80.24	0.722	0.68	0	0.762	82.15	0.751	0.701	0	0.733
CV-ACM [21] 2014	82.36	0.761	0.712	0.261	0.673	83.6	0.78	0.729	0.243	0.641
*K*-means [47] 2015	73.3	0.701	0.671	0	0.78	75.2	0.72	0.69	0	0.745

## Data Availability

Dataset 1: the data provided in this publication will be available from the corresponding author upon request. Dataset 2 is freely available online. The link is provided in the reference.
